# *CFEL–ASG Software Suite* (*CASS*): usage for free-electron laser experiments with biological focus^1^

**DOI:** 10.1107/S1600576716009201

**Published:** 2016-06-23

**Authors:** Lutz Foucar

**Affiliations:** a Max Planck Institute for Medical Research, Jahnstrasse 29, Heidelberg, 69120, Germany

**Keywords:** free-electron lasers, FELs, data analysis, femtosecond crystallography, SFX, single-particle experiments, pump–probe techniques, fluorescence, computer programs

## Abstract

*CASS* [Foucar *et al.* (2012). *Comput. Phys. Commun.* **183**, 2207–2213] is a well established software suite for experiments performed at any sort of light source. It is based on a modular design and can easily be adapted for use at free-electron laser (FEL) experiments that have a biological focus. This article will list all the additional functionality and enhancements of *CASS* for use with FEL experiments that have been introduced since the first publication. The article will also highlight some advanced experiments with biological aspects that have been performed.

## Abstract 2

An overview of how the well established *CFEL–ASG Software Suite* (*CASS*) can be used for serial femtosecond crystallography data is given.

## 1. Introduction   

Free–electron lasers (FELs) are fourth-generation light sources that deliver ultra-short, coherent and intense pulses of radiation extending from the vacuum ultraviolet to the X-ray regime. They allow the investigation of a plethora of phenomena in biology that were not accessible using conventional synchrotron radiation sources, since radiation damage limits high-resolution imaging of biological material. In the case of X-ray crystallography, the most successful method for high-resolution structure determination to date, this poses limitations on the size of the crystals that can be analyzed, since lowering the incident fluence to reduce damage calls for larger crystals to sustain sufficient diffraction intensity. In many cases, however, growing large crystals is very difficult, whereas micrometre-sized crystals are observed frequently. X-ray FELs exceed the peak brilliance of conventional synchrotrons by almost 10 billion times. They promise to break the nexus between radiation damage, sample size and resolution by providing extremely intense femto­second X-ray pulses that pass through the sample before the onset of significant radiation damage. This concept of ‘diffraction before destruction’ has been demonstrated at the Linac Coherent Light Source (LCLS), the first operational hard X-ray FEL, for tiny protein crystals (Chapman *et al.*, 2011 ▸) and single mimivirus particles (Seibert *et al.*, 2011 ▸). Moreover, it has been shown that high-resolution high-quality diffraction data (Boutet *et al.*, 2012 ▸) can be obtained from micrometre-sized crystals by FEL-based serial femtosecond crystallography (SFX).

As well as the structure of biological samples, it has become possible to study their functionality on an ultra-short timescale using pump–probe techniques (Aquila *et al.*, 2012 ▸; Barends, Foucar *et al.*, 2015 ▸; Pande *et al.*, 2016 ▸).

### 1.1. *CASS* at FELs   

**Figure 1:**
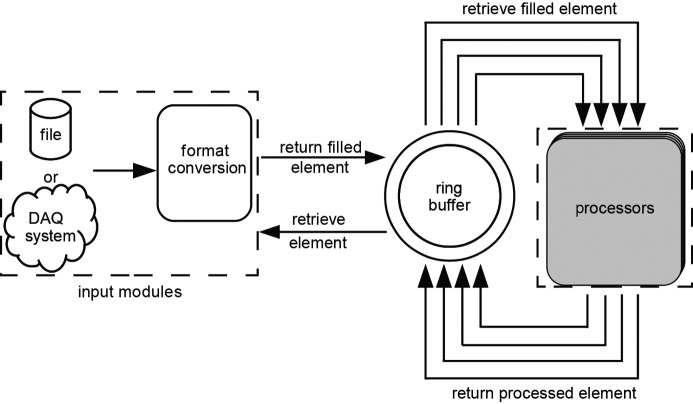
Overview of the general layout of *CASS*. The arrows mark the flow of the CASSEvents. Adapted from Foucar *et al.* (2012). The major difference is that the processing part is no longer split up into pre- and postprocessing units.

The *CFEL–ASG Software Suite* (*CASS*) was originally created to view, process and analyze multi-parameter experimental data acquired at FELs using the CFEL-ASG Multi-Purpose (CAMP) instrument (Strüder *et al.*, 2010 ▸). To allow *CASS* to be used at multiple different FEL facilities, a modular design was chosen that separates the input part from the processing part, coupling them by a ring buffer (Fig. 1 ▸). Because the processing part of *CASS* itself is also based on a modular design, it can easily be adjusted to various experiments that are conducted with the CAMP instrument and its successor, the LAMP instrument (Roberts, 2014 ▸). The modular design requires that the different modules pass information between each other. The result of a module is stored in either zero- (0D), one- (1D) or two-dimensional (2D) containers (Foucar *et al.*, 2012 ▸), which were previously called histograms and now are simply called results, and can be used as input for a module that follows in the analysis chain.

One of the most attractive aspects of *CASS* is that it can be used both ‘on-line’, using a live data stream from the free-electron laser facility’s data acquisition (DAQ) system to guide the experiment, and ‘off-line’, on data acquired from the experiment at a later time.

*CASS* has been used successfully in a variety of different experiments (Andreasson *et al.*, 2014 ▸; Barends, White *et al.*, 2015 ▸; Barends, Foucar *et al.*, 2015 ▸, 2014 ▸, 2013 ▸; Barty *et al.*, 2012 ▸; Boll *et al.*, 2013 ▸, 2014 ▸; Botha *et al.*, 2015 ▸; Boutet *et al.*, 2012 ▸, 2015 ▸; Bublitz *et al.*, 2015 ▸; Chapman *et al.*, 2011 ▸; Erk *et al.*, 2014 ▸, 2013*a* ▸,*b* ▸; Frasinski *et al.*, 2013 ▸; Fukuzawa *et al.*, 2013 ▸; Gomez *et al.*, 2014 ▸; Gorkhover *et al.*, 2012 ▸; Johansson *et al.*, 2012 ▸; Küpper *et al.*, 2014 ▸; Kassemeyer *et al.*, 2012 ▸; Koopmann *et al.*, 2012 ▸; Loh *et al.*, 2013 ▸, 2012 ▸; Lomb *et al.*, 2011 ▸; Martin, Loh *et al.*, 2012 ▸; Martin, Wang *et al.*, 2012 ▸; Motomura *et al.*, 2013 ▸; Mucke *et al.*, 2014 ▸; Nass *et al.*, 2015 ▸; Park *et al.*, 2013 ▸; Pedersoli *et al.*, 2013 ▸; Rudek *et al.*, 2013 ▸, 2012 ▸; van der Schot *et al.*, 2015 ▸; Seibert *et al.*, 2011 ▸; Starodub *et al.*, 2012 ▸; Stern *et al.*, 2014 ▸; Yoon *et al.*, 2011 ▸; Zhaunerchyk *et al.*, 2013 ▸), including the aforementioned single-particle and the emerging SFX experiments. Moreover, it has been used for experiments at different FEL facilities such as LCLS, SACLA and FLASH.

In order to exploit the full capabilities of *CASS* for experiments with a biological focus, additional features and enhancements needed to be implemented, which will be discussed in this paper.

## 2. *CASS* for SFX/SAXS/single-particle imaging   

For most experiments with a biological focus, the data analysis flow involves applying essential corrections to the raw recorded data, then extracting the data of interest, with subsequent treatment of the retrieved information. The latter depends on the type of experiment and involves programs such as *CrystFEL* (White *et al.*, 2012 ▸, 2016 ▸), *nXDS* (Kabsch, 2014 ▸) and *GIPRAL* (Kassemeyer *et al.*, 2013 ▸). *CASS* is used for the first step, and applies all necessary corrections to the raw data and extracts the necessary information for the subsequent processing steps. This involves being able to provide the data in the format that programs such as *CrystFEL* or *nXDS* expect. Moreover, as each experiment is different, the various user-defined parameters such as those for the peak finding algorithms (see below) used in crystallographic experiments must be carefully adjusted for each experiment, and in many cases for each sample.

The majority of the already existing modules can be used for experiments driven by biological questions. Most of the basic functionality of analysing and processing data from pixel detectors, such as determining the noise and offset (sometimes called ‘pedestal’) of the detectors and removing common mode fluctuations, can be reused for these kinds of experiments. However, some of the most important feedback to determine the success of an experiment can only be obtained by adding new or modifying existing functionality within *CASS*.

Most of the missing functionality can be added by creating new or more specialized processing modules (so-called ‘Processors’) to *CASS* (which were called ‘PostProcessors’ in previous versions of *CASS*). Some of the required additional features could only be implemented by rewriting and enhancing the existing viewer for *CASS*, called the *JoCASSViewer*.

The changes that have been implemented are listed in the following sections.

### 2.1. HDF5 output   

Programs such as *CrystFEL* and *GIPRAL* expect the input data to be in the HDF5 file format (The HDF Group, 2016 ▸) with a given structure. *GIPRAL* expects for each event of interest a single file containing all information that describes this event, whereas the latest versions of *CrystFEL* also allow the use of multi-event HDF5 files. To be able to write HDF5 files with any group structure, the already existing module that saved certain data into the HDF5 format, with the possibility to generate multi-event HDF5 files, has been rewritten to allow the user to place the data into a user-defined data group within the HDF5 file. With these changes it is also possible to define the name of the Processor that contains the 0D, 1D or 2D result to write, and provide a folder into which the data should be stored. If desired, the name of the recorded data can be different from the name of the Processor. If no alternative name is provided the name of the Processor is used.

Since the file system response time is slower when many files are stored in a single folder, an option has been introduced that enables the creation of sub-directories with a user-defined number of files. This leads to a speed-up when generating the list of files needed in the subsequent processing steps.

In order to reduce the disk space occupied by the written data, the Processor has an option to compress the datasets stored within the HDF5 files, which is achieved by using the zlib compression capability provided by the HDF5 library.

### 2.2. CBF output   

Recently, the developer of *XDS* (Kabsch, 2010 ▸) has enhanced his well established crystallographic software suite to work with data taken at FELs using SFX-like experiments. The new program suite is called *nXDS* (Kabsch, 2014 ▸). This program suite expects the input data to be in the crystallographic binary file (CBF) format.

To be able to use *nXDS* for the analysis steps following the *CASS* treatment of the data, a module has been added to *CASS* that allows the 2D results to be stored in the CBF format. The 2D data are written using the compressing ‘x-CBF_BYTE_OFFSET’ technique (Ellis & Bernstein, 2005*a* ▸,*b* ▸). Because *CASS* internally stores the data as floating point variables, they are converted to signed 32 bit integers before they are written to the CBF format.

In some cases *nXDS* expects the raw untreated data of the detector as input and will apply all necessary corrections to the data itself. Therefore it is possible to store the untreated data with only bad-pixel marking to CBF. The offset that has been calculated from the calibration runs is written by the Processor at the end of the analysis into a separate CBF file. This file can optionally be used as the ‘DARK_CURRENT_IMAGE’ within *nXDS*.

No additional information about the experiment, for example the photon energy, the detector distance or pixel size, is extracted from the raw recorded data and added to the CBF file as this information is ignored by *nXDS*.

### 2.3. Data layout   

SFX, small-angle X-ray scattering (SAXS) and some single-particle experiments at LCLS are carried out using the CSPAD (Hart *et al.*, 2012 ▸) detector. Unlike in traditional pixel detectors used for biological diffraction experiments, the data from this detector are not provided to the user in the laboratory frame of reference (lab frame). The rearrangement of the data has to be done by the user and is essential for quick visual inspection of the data in SFX experiments, for the interpretation of SAXS data and for all subsequent analysis steps in single-particle experiments. There are many different standards that define the conversion of the data from the raw data layout into the lab frame. Since the SFX data analysis is tightly integrated with the use of *CrystFEL*, the natural choice is using the *CrystFEL* detector geometry description file (White *et al.*, 2012 ▸) standard, which defines where pixels of the raw data are to be placed in the lab frame with the help of a text file, the so called ‘*CrystFEL* geometry file’.

For this purpose a module that parses the *CrystFEL* geometry file and uses the information to generate a new frame where the pixel positions correspond closely to their positions in the lab frame has been developed. Using this new frame, it is easy to generate a virtual powder pattern which helps to estimate the relative amount of data collected so far and the maximum expected resolution of the recorded data.

### 2.4. Crystal hit finder   

Many of the recorded detector frames do not contain any useful data for SFX or single-particle experiments, for instance because no sample was present in the interaction region when the FEL beam was on. These events are called ‘empty’ events. Only when the beam intersected the investigated sample are the data useful for further analysis. For data reduction and speed-up it is essential that only frames that contain potentially valid data are used. In SFX-type experiments ‘empty’ frames can be distinguished from the data-containing frames (so-called ‘hits’) by looking for the presence of Bragg spots or reflections within the frame. A specialized module that analyzes the frame and adds all potential Bragg spots to a list has been implemented, based on an algorithm presented by Zhang *et al.* (2006 ▸). This analysis works as follows.

First, the algorithm searches for a pixel that is above a user-selected threshold. If this pixel has the highest value of all pixels within a user-defined box, with the pixel being in the center of the box, then the mean value and the standard deviation of all pixels within the box are calculated. Otherwise the algorithm continues to search for pixels above the threshold. Subsequently, the signal-to-noise ratio is calculated for all pixels within the box. To determine the mean and standard deviation of the background pixels, the following technique is chosen: If a pixel’s signal-to-noise ratio exceeds a user-provided threshold it is removed from the distribution and the statistical values per pixel are recalculated. This procedure is repeated until none of the remaining pixels exceeds the user-set signal-to-noise-ratio.

Once the mean and standard deviation of the background pixels have been determined, the signal-to-noise-ratio of all the pixels within the box is calculated. Next, the algorithm finds and counts all pixels that exceed another user-set signal-to-noise ratio and are directly or indirectly connected to the central pixel. If the count exceeds a set threshold the found peak is regarded as a Bragg peak. Values like the centroid position, the central peak index, the highest pixel value, and the summed intensity of all pixels contributing to the Bragg peak are then calculated and put into a table.

**Figure 2:**
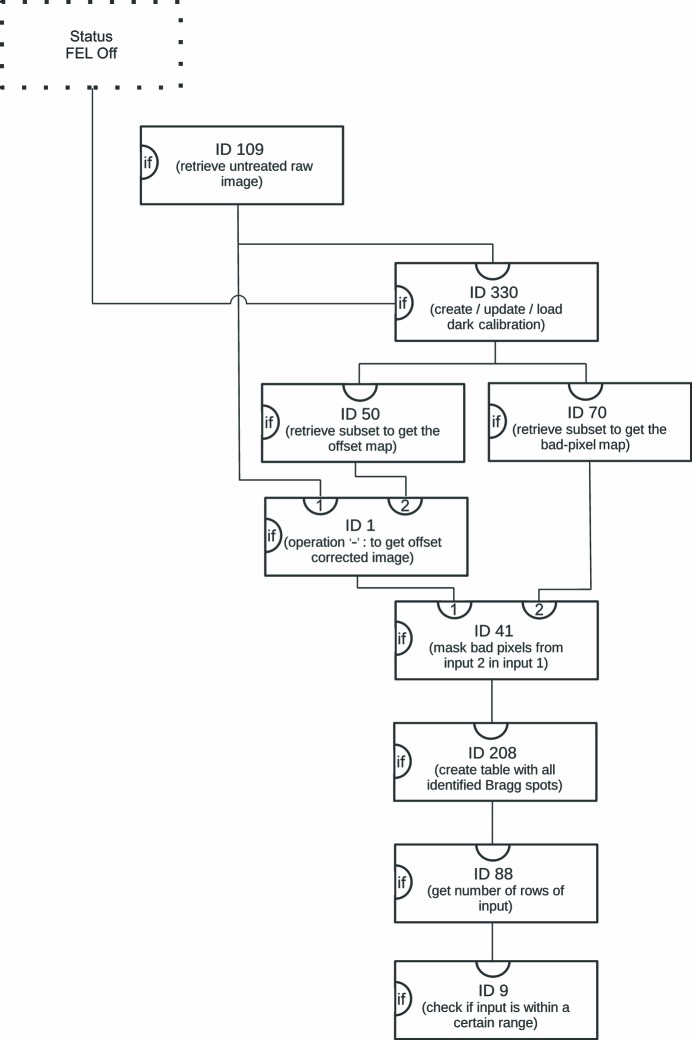
Diagram to visualize that part of the .ini file that is responsible for identifying whether the current event is a hit. Details are given in §2.4. The boxes are individual Processors with the indicated ID. The parentheses contain a short description of the purpose within the context. The dotted box indicates the part of the .ini file that determines the FEL status. A line into the ‘if’ input of the box indicates that the Processor will only be run conditionally. A line into the top input indicates the input and the line going out at the bottom of a box represents the result of the Processor.

Another Processor evaluates the number of rows of the table and thus returns the number of potential Bragg peaks within the frame. This number is evaluated in another module that checks if it is within a user-selected range. If this is the case, the event is labeled as containing a hit (see also Fig. 2 ▸).

Using the information provided in the table the ratio of saturated to non-saturated Bragg reflections can be determined. This is achieved by using a Processor that returns a sublist of the table, based on the highest pixel value being above a user-set threshold. By evaluating the size of this sublist and using the module to divide the two numbers, the ratio can be retrieved. The latter values are put into a histogram for graphical feedback.

It is important to realize that the values for the threshold and the box size in the hit finding module strongly depend on experimental parameters such as crystal size, beam intensity *etc*. Thus, these parameters need to be checked for each sample and experiment, but rarely change during an experiment owing to the nature of the algorithm.

### 2.5. Radial average   

For SAXS experiments it is necessary to evaluate the radial dependence of the detector data. With the already existing modules, the radial average of a frame would be obtained by using a Processor that outputs the data into the lab frame and then using a module to convert the resulting image to polar coordinates and project the result onto the radius axis. Owing to the gaps between the tiles of the CSPAD detector, this would lead to peaks and nodes in the result that do not originate from the experiment but from the data analysis itself. Therefore a specialized Processor to calculate the radial average was introduced.

Using the *CrystFEL* geometry file this specialized Processor calculates the radial value of each pixel, *i.e.* its distance to the direct beam. Together with the information about the current wavelength and the detector distance, the *q* value for each pixel is evaluated using the equation
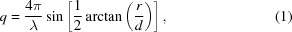
where λ is the photon wavelength, *r* is the radius and *d* is the detector distance. The calculated *q* values are put into a histogram where the corresponding pixel value is used as weight. The bins of the histogram are then normalized by the number of entries per bin.

### 2.6. Single-particle experiments   

In typical single-particle experiments it is essential to separate frames containing data (‘hits’) from the ‘empty’ ones. This process is called ‘hit finding’. Hit finding in single-particle experiments is different from the process in SFX experiments. Andreasson *et al.* (2014 ▸) proposed an algorithm that allows the detection of non-empty frames, when the number of frames containing data is much less than the number of empty frames. This algorithm can easily be implemented in *CASS* using the existing modules.

Once the non-empty frames have been identified, it is necessary to distinguish frames containing single-particle data from those containing a cluster of multiple single particles. This information is needed as feedback to determine whether the sample delivery has to be modified. To categorize the recorded data into these two different ensembles, Processors that retrieve the autocorrelation of the frame have been implemented. Three different approaches to determine the autocorrelations were realized. The first was to retrieve the autocorrelation in Cartesian coordinates, the second to return the autocorrelation in polar coordinates and the third to calculate the fast Fourier transformation (FFT) of the frame.

Besides minimizing the occurrence of clusters of single particles, it is crucial to keep the size distribution of the particles under investigation small. The currently available algorithms that retrieve a three-dimensional model of the single particle such as *GIPRAL* (Kassemeyer *et al.*, 2013 ▸) rely on the fact that the difference in the recorded frames is simply given by taking ‘snapshots’ of an object at different angles. The objects themselves should be identical. Therefore, a broad size distribution will result in major difficulties for the three-dimensional model retrieval. A value that is proportional to the size of the recorded single particle can be calculated using the radial average of the image, and then the distance between the first two nodes of the radial average can be determined. Therefore, a Processor that will extract the distance between the first two nodes of a one-dimensional trace was added to *CASS*. By histogramming the determined distance, the uniformity of the sample can easily be determined.

### 2.7. *JoCASSViewer*   

One of the viewers available in *CASS*, the *JoCASSViewer*, is based on the Qwt library (Rathmann & Wilgen, 2016 ▸) which provides widgets for technical applications based on the Qt library (The Qt Company, 2016 ▸). *JoCASSViewer* has been completely rewritten to be compatible with the latest version of the Qwt library and to be able to read other data formats than the result data format of *CASS*. With the new version it is possible to read and store data in the histogram, HDF5 and CBF file formats.

In the course of this rewrite, the display of 2D results has been enhanced to optionally put data in the raw data layout into the lab frame and display the diffraction resolution of each pixel on request (see below). To enable this one has to provide information about the transformation from the raw data layout to the lab frame data in the form of the *CrystFEL* geometry file. This is used to assemble a new frame where the pixels correspond to their position in the lab frame. Given the transformation information, the detector distance and the wavelength, the diffraction resolution corresponding to each pixel’s distance from the direct beam can be calculated using the following equation:
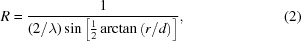
where λ denotes the wavelength in ångström, *r* the distance of the pixel from the direct beam and *d* the detector distance in metres. A box close to the cursor displays the resolution at the cursor position.

To be able to easily identify saturated pixels, a special color scale was introduced that allows the visualization of pixels that have values that lie outside a user-selected range. All pixels with values that lie within the user-selected range are displayed in white. Pixels having higher values are displayed in red and pixels with lower values in black.

### 2.8. Tools to work with *CASS* for SFX   

Using *CASS* allows one to get real-time feedback about important parameters of the experiment such as the hit rate or pixel saturation. However, in order to get feedback about the data quality, for example, in SFX experiments, such as the indexing rate, it is necessary to run other programs on the data extracted by *CASS*. In most experiments such feedback is needed as soon as possible. Therefore, running these programs as fast as possible is essential for maximizing the chances of obtaining a good dataset and making the most use of the limited time available at FEL sources. One way to speed up the analysis in the processing chain is to parallelize the required tasks as much as possible, which takes advantage of any available computer clusters. To ease the process of generating the input files necessary to run *CASS* and the succeeding analysis programs in parallel, multiple bash scripts have been developed.

The first step in the analysis chain is to run *CASS* on the raw data and extract the events of interest. The fastest way to analyze the raw data in cases where each event is self-explanatory is to parallelize the analysis as much as possible by splitting up the raw data into portions of data called ‘slices’ that can be analyzed independently. Each of these analyses is called a job. For each job, the user has to provide *CASS* with the list of files that should be analyzed and an instruction file, called a .ini file, which tells *CASS* how to analyze the data. To be able to add this job to the computer cluster for processing, a batch script has to be prepared that contains the information about the location of the *CASS* binary, the file containing the list of files to be processed and the .ini file. The generation of these files is automated by using a bash script. By providing information about which runs should be processed in combination with other runs, such as a dark-calibration run, the script prepares all necessary files to be able to submit the job to the cluster queue. This information is provided in a comma-separated format (csv) file. To ease the user experience, in future the comma-separated file can be modified *via* a multi-user HTML interface, which also allows control over the analysis jobs on the cluster.

As *CASS* is highly modular, the output for the subsequent analysis steps can easily be adjusted to upcoming new requirements by providing a new Processor that outputs the results in the requested format.

Once the events containing the data of interest have been extracted from the raw data, generally in SFX experiments *CrystFEL* is run to determine the indexing rate, providing the first feedback about the data quality. Though *CrystFEL* itself is capable of processing multiple events in parallel, experience shows that by running multiple instances of *CrystFEL* in parallel one can get a speed-up of this step in the analysis chain compared to running just one instance of *CrystFEL* on the data of one run. *CrystFEL* needs to know the files it has to process and how to process them. The list of files it should process is provided in a text file, while the options telling *CrystFEL* how the data should be analyzed are provided as command line parameters. The script that parallelizes the *CrystFEL* task generates the list of all files that belong to the run. It then splits this list into multiple lists which contain subsets of the whole list with a user-selected maximum number of files. For each of the subsets the command line parameters for *CrystFEL* are generated from a template and put into a bash script for the computer cluster. Information on which template to use for which run is provided in a csv file, making it the central control for all bash scripts. This system has been successfully applied in multiple SFX studies (*e.g.* Barends, Foucar *et al.*, 2015 ▸, 2014 ▸, 2013 ▸; Barends, White *et al.*, 2015 ▸; Nass *et al.*, 2015 ▸; Botha *et al.*, 2015 ▸; Boutet *et al.*, 2015 ▸).

## 3. Changes in *CASS* not related to bio-driven experiments   

The processing analysis scheme described in the first *CASS* paper (Foucar *et al.*, 2012 ▸) had one major drawback. A significant amount of memory was required since each Processor needed to store a large number of results in order to reduce the possibility that the result of a particular event was overwritten with data of another event, before all Processors that depend on the result had finished processing that particular event. In principle, this could happen regardless of whether *CASS* was running in blocking or in non-blocking mode. This led to a memory usage of up to 10 gigabytes per *CASS* instance. While memory is cheap these days, it is still not advisable to use most resources of the computer. Therefore, the way that the results are handled by the individual Processors has been updated. Each call to a module will initiate the calculation of the result for a specific event. The result is then locked and cannot be overwritten. After the Processor manager has passed the current event to all subsequent Processors, the result of the current event is released. This scheme removes the need to store enough results to minimize the possibility that another event overwrites the result of an already processed event.

To remove unnecessary checks, the Processor manager rearranges the analysis chain during the setup such that Processors that depend on the result of another module are called after their dependencies.

To achieve a better structure within the core processing sequence and to make it easier for developers to add novel functionalities, different types of modules have been introduced. In general there are four different types of tasks during *CASS* processing.

(1) Processing an event independently of the results of other events.

(2) Accumulation of the results from other Processors.

(3) Providing constant values that do not change during the course of the analysis.

(4) Purely operational tasks that do not return a result, *e.g.* quitting *CASS*.

A new general Processor and a new accumulating Processor were introduced. While the general Processor manages multiple independent results, ensuring that none of the results will be overwritten while the current event is still being processed, the accumulating Processor contains just one result, which is updated once for each event. The modules returning constant values are based on the general Processor where the part that should evaluate the event does nothing. The operational functions such as writing results are realized using the general Processor, where the virtual functions that return the result of a computation and create the managed list of results are overwritten to generate errors. This ensures that *CASS* will report erroneous use, when the user tries to use those Processors in an unforeseen way.

In previous versions of *CASS*, all results had to be of a predefined size. The new version of *CASS* introduces table-like results, which can have a variable number of rows. Functionality that had to be hidden from the user through helper classes and highly specialized Processors, such as for the analysis of data from delay-line detectors, can now easily be generalized. Being able to return results in a table allows easier interactions with the data and removes the rigidity of the analysis imposed on the user by the highly specialized modules. This allows the processing part of *CASS* to be considerably leaner and therefore more supportable. An example that makes use of this new functionality is the new module that identifies potential Bragg peaks within an image. Additional modules have been added to allow operations such as retrieving rows that fulfill a certain condition and retrieving a specific column of the list. Porting the specialized functionality to the more general table Processor approach is still ongoing.

### 3.1. Enhanced dark calibration   

Since *nXDS* and *CrystFEL* need to have the detector data corrected in different ways (*nXDS* needs raw data with only the bad pixels set to negative values, whereas *CrystFEL* needs dark-calibrated data with bad pixels set to 0), it was obvious that the dark calibration needed to be decoupled from the detector data retrieval. Therefore a new Processor was added to *CASS* that allows the dark-calibration maps to be calculated from the raw data. One can then use these maps together with other modules to produce the calibrated detector image. For instance, one can use the Processor with ID 1 to subtract the offset map from the raw data to get the dark-calibrated images that *CrystFEL* needs as input for indexing and integration.

This newly added Processor has ID 330 and takes the raw detector images as input. It has two modes for processing the input images. The first is called training mode, in which a user-selected number of images are stored in internal memory. From the stored images a pixel-wise mean and standard deviation of the mean (from now on called stdv) are determined, while pixels that are outliers of this distribution are disregarded. The mean value corresponds to the offset value and the stdv to the noise value in the dark calibration. The number of images that contributed to the calculation of the offset and the noise value is recorded for each pixel. In the second mode, new images are added to the offset and noise maps. The user has the option whether this should be done in a cumulative way, where all images have the same weight. It is known that owing to temperature instabilities, which are caused by the type of signal that the detector records, the offset and noise values will change over time. Therefore, the user can choose to add the new images in an ‘exponential moving average and standard deviation’ way, where the last added images contribute more to the resulting values than images that were added earlier. This allows the temperature-induced changes of the detector to be more closely followed. In both of these options any pixel that is an outlier is not added to the calculation of the maps. Outliers are determined using the following equation:
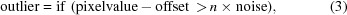
where *n* is a user selectable value.

The resulting offset and noise maps, together with the counts, are vertically stacked and stored in a two-dimensional result. Using the Processor with ID 70 one can separate the different maps within *CASS* for further calibration of the raw detector data. To continue working with the written maps with the old way of dark-calibrating the detector images, these maps can be written to a file in the *CASS* format at the end of the training and in a user-selectable update period. If no filename is provided to the Processor, a file name will be automatically determined from the name of the Processor and the date and time. A link to the latest generated dark-calibration file is generated from the Processors name alone.

After the training and before the update step, each pixel is checked to determine if it is in a bad or broken state. A pixel is bad when its offset and noise values are not within user defined, or automatically determined, boundaries. The automatic determination of the boundaries for both the noise and the offset values is achieved by evaluating the mean and stdv of the mean of the offset and noise values in the maps using the following equations:
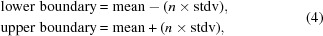
where *n* is a user-selectable value. Outliers from the distribution of offset and noise values are removed during the determination of the mean and stdv. If the pixel’s offset and noise values are within the boundaries the corresponding value in the mask is set to 0, and otherwise it is set to 1. This mask is also vertically stacked in the resulting two-dimensional histogram output. One can use this mask in the Processor with ID 41 (Table 1 in Appendix *A* [App appa]) to set all masked pixels to a user-defined value.

To provide the offset and noise maps to the subsequent *CASS*-based analysis even when no training and updating is performed, the Processor is able to read the dark-calibration file in the *CASS* format. Because only the offset and noise values are stored within the dark-calibration file, the counts have to be artificially set to training size. The mask is generated as described above after the offset and noise maps have been initialized with the values from the dark calibration file.

With these automated adjustments of parameters, it is not usually necessary to adjust these parameters by hand during an experiment.

## 4. Examples   

The described modularity of *CASS* makes it relatively easy to adapt to various different types of experiments. Two examples will be outlined in this section: an experiment whereby the onset of damage in an SFX experiment was investigated (to be published) and a pump–probe experiment where, for the first time, ultra-short time delays were used to visualize the motions of myoglobin. Apart from the specific requirements of these experiments, which will be detailed in the following sections, feedback on the hit rate, which is an indication of the successful interaction of the FEL light with the sample, and amount of data of the experiment is required for all such experiments.

**Figure 3:**
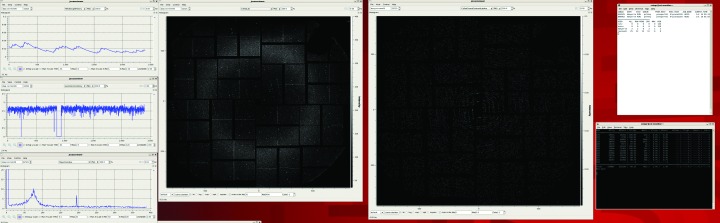
Screenshot taken while collecting data from a damage experiment at the CXI endstation at LCLS. Data are displayed using the *JoCASSViewer*. The top-left image shows the current hit rate. The picture below displays the pulse energies of the last 2400 shots. And the bottom-left image shows the average fluorescence spectrum of the investigated target. To the right of these images is the most recent identified hit, and next to it an average image of all found hits (virtual powder pattern).

The screenshot in Fig. 3 ▸ shows a typical real-time (‘online’) *CASS* output, which was used as live feedback during the data acquisition. The top-left image shows the current hit rate. It is calculated as the ratio of frames containing diffraction data as identified by the *CASS* hit finder (hits) to empty frames. In this example, the ratio of hits *versus* empty frames over the last 2400 FEL shots is displayed. The middle left picture displays the pulse energies of the last 2400 shots. This information helps to identify whether there are any problems with the FEL source. The bottom left image shows the average fluorescence spectrum of the target under investigation, which will be described in more detail in the following section. To the right of these images is a display of the detector output with the most recently identified hit and, next to this, an average image of all hits found (a virtual powder pattern). The virtual powder pattern can be used as an indication of whether enough data for the dataset have been collected so far. It also serves as a good indication for flow-aligned samples, since these result in powder patterns that are not radially symmetric, whereas radially symmetric rings are observed when no flow alignment occurs.

### 4.1. Studying radiation damage in protein crystals   

**Figure 4:**
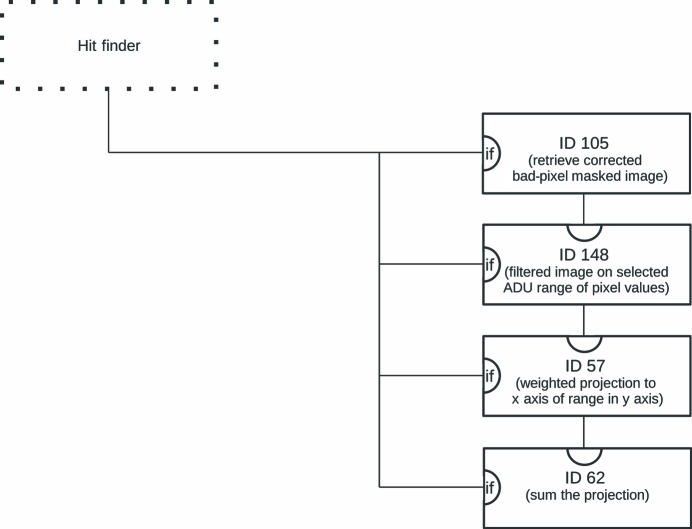
Diagram to visualize that part of the .ini file that is responsible for extracting and processing the fluorescence information. Details are given in §4.1. The boxes are individual Processors with the indicated ID. The parentheses contain a short description of the purpose within the context. The dotted box indicates the section of the .ini file that determines whether the current image is a hit (see also §2.4). A line into the ‘if’ input of the box indicates that the Processor will only be run conditionally. A line into the top input indicates the input and the line going out at the bottom of a box represents the result of the Processor.

In July 2013 ▸ an experiment (Nass *et al.*, 2015 ▸) was performed at the CXI endstation at LCLS (Liang *et al.*, 2015 ▸), which was aimed at understanding the mechanism that leads to target sample damage during interaction with the FEL beam. In addition to identifying the FEL shots that contained diffraction data from the empty frames, the sample fluorescence was also under investigation in this experiment. A dedicated detector system based on the design described by Alonso-Mori, Kern, Sokaras *et al.*( 2012 ▸) was introduced. This allowed the X-ray fluorescence to be monitored during the interaction of the target and the FEL beam in the range of 7.04–7.17 keV, thereby providing the possibility to correlate the fluorescence information with the diffraction by the target. The difficulty was extracting the fluorescence information from the dedicated detector. Alonso-Mori, Kern, Gildea *et al.* (2012 ▸) described in detail the steps to be taken in order to properly extract the fluorescence data from the detector. This technique was accomplished by creating a histogram of the analog to digital unit (ADU) value for each pixel of the offset and gain-corrected detector data in the area of interest. These histograms were stored in an HDF5 file and then further processed by using Python scripts to extract the final fluorescence spectra. However, this can only be performed once the data have been recorded. In order to achieve live feedback during data acquisition the following analysis chain was built within *CASS* (for a visual representation, see Fig. 4 ▸):

(*a*) retrieve offset and gain-corrected detector data for all pixels whose ADU values are in the range of 18–50 ADU and the other pixel values are set to zero

(*b*) project the pixels in the range of interest onto the *x* axis, with a normalization to the number of pixels included in each column during the projection

Using this chain the average fluorescence spectrum could be displayed (Fig. 3 ▸). This was sufficient to judge the success of the experiment during the live data acquisition even though the photon-energy axis was not yet calibrated.

### 4.2. Observing ultrafast functional motions in a biomolecule   

**Figure 5:**
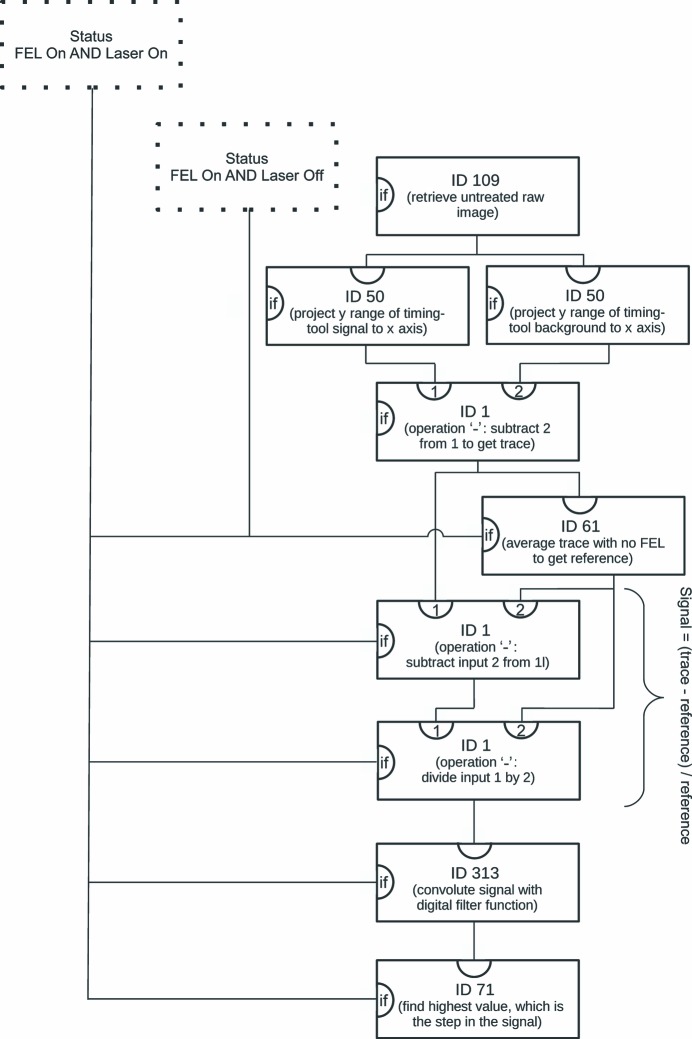
Diagram to visualize that part of the .ini file that is responsible for extracting the delay between the FEL and laser. Details are given in §4.2. The boxes are individual Processors with the indicated ID. The parentheses contain a short description of the purpose within the context. The dotted boxes represent the parts of the .ini file that determine the FEL and laser status. A line into the ‘if’ input of the box indicates that the Processor will only be run conditionally. A line into the top input indicates the input and the line going out at the bottom of a box represents the result of the Processor.

In summer 2014 an experiment was performed at the CXI endstation of the LCLS, which was aimed at the investigation of the collective atomic motions of myogolobin upon ligand dissociation on an ultra-short timescale. The findings were reported by Barends, Foucar *et al.* (2015 ▸). The reaction was initiated using 532 nm laser light produced at the output of an optical parametric amplifier which was fed by a Ti–sapphire laser pumped with a frequency-doubled neodymium-doped yttrium lithium fluoride laser. The induced structural changes were probed using the FEL beam. Since the timing between the laser and the FEL differed from shot to shot by as much as 250 fs because of the inherent jitter of the FEL, a so-called ‘timing tool’ (Bionta *et al.*, 2011 ▸) was added to the experimental setup to measure the actual time delay between the laser and the FEL. The technique that allows extraction of the exact timing between the laser and the FEL is described in detail by Bionta *et al.* (2011 ▸). In *CASS*, the data from the timing tool were assessed to extract the timing between the fs laser and the FEL beam as follows (for a visual representation see Fig. 5 ▸):

(*a*) Horizontally project the signal-containing part of the timing tool image and subtract the local background, which is a projection close to the signal-containing part.

(*b*) Subtract a reference signal that contains the signal from the laser-generated white light without the FEL from the current signal and divide by the reference.

(*c*) Convolute the resulting signal with a digital filter signal that was provided by the LCLS DAQ team.

(*d*) Find the highest point within the convoluted signal, which identifies the pixel that corresponds to the position in the spectrum where the drop occurred.

Using this analysis chain it was possible to correlate each shot that contained valid diffraction information with the correct corresponding delay between the laser and the FEL.

## 5. Performance   

Running in online mode at the LCLS, *CASS* can access only one of the provided monitoring feeds. In a typical scenario at the CXI endstation there are five feeds where each gets 1/5th of the 120 Hz full rate. Therefore, in online mode *CASS* needs to be able to handle 24 Hz, which in a typical SFX experiment is no problem on the monitoring computers that LCLS provides. These monitoring nodes consist of two Intel Xeon CPU ES645 @ 2.4 GHz processors with six logical (hyper-threading) cores each.

At the time of writing of this manuscript it was only possible to get a 30 Hz data rate at SACLA, where one is provided with a computer node that can access the online data feed. In a typical SFX experiment, where only hit-finding capabilities are needed, *CASS* can keep up with the provided 30 Hz.

## 6. Availability   

*CASS* is developed under the terms of the GNU General Public License, version 3, as of 29 June 2007. The latest stable version can be publicly downloaded from https://github.com/foucar/CASS. The documentation for the latest stable version can be found at http://www.mpi-hd.mpg.de/personalhomes/gitasg/cass/.

## Figures and Tables

**Table 1:** General operations

Number	Purpose
00001	User-selectable operation on two results
00002	Operation on result with value or 0D result
00004	Apply boolean NOT to 0D result
00009	Check whether result is in a range
00012	Constant value
00013	Identity operation (returns the input)
00015	Check whether value of 0D result has changed
00040	Threshold result
00041	Set bin value in result to user value, when corresponding bin value of another result is in given range
00050	Project 2D result onto *a* axis
00057	Weighted projection of 2D result onto *a* axis
00051	Integral of 1D result
00055	Rotate, transpose or invert axis on 2D result
00056	Contains the result of the previous event
00060	Histogram 0D, 1D or 2D results to a 1D result, optionally with custom weights and remembering the counts per bin
00061	Averaging of results
00062	Summing of results
00064	Add values of 0D, 1D or 2D results into 1D result (append on right end, shifting old values to the left)
00065	Histogram two 0D results to a 2D result
00069	Use two 0D results for a scatter-plot-like 1D result
00070	Subset a result
00071	Retrieve a user-selectable value of a result
00081	Retrieve a user-selectable bin of a 1D result
00072	Returns a user-selectable column from a table-like 2D result
00073	Returns a subset of a table-like result based on a condition on a selectable column
00074	Returns a specific value of a specific row of a table-like result
00079	Create 2D result from two columns of a table with optional weight column
00075	Clear a result
00076	Quit *CASS* when condition is met
00077	Check if eventid is on a user-provided list
00078	Count how many times it has been called (counter)
00082	User-selectable statistics value of all values of a result
00085	FWHM for a peak in a given range of 1D result
00086	Find step in a given range of 1D result
00087	Find center of mass in a given range of 1D result
00088	Retrieve an axis parameter of the result
00089	High or low pass filter on 1D result
00090	*q* average of detector image
00091	Find local minima or maxima in a 1D result
00202	Transform 2D result from Cartesian coordinates to polar coordinates (interpolating)
00301	Median over last values
00313	Convolute a result with a kernel
00312	FFT of a 1D result
01602	Rearrange 2D result raw data using a geom file

**Table 2:** Data retrieval Processors

Number	Purpose
00109	Retrieve raw untreated pixel detector image
00120	Beamline data
00121	Eventcode check
00122	Eventid retrieval
00123	Beamline spectrometer data
00130	EPICS data
00110	Acqiris waveform
00230	Photon energy of shot
00405	Pulse duration of shot

**Table 3:** Output Processors

Number	Purpose
01002	Put selectable results into HDF5 files
01500	Put a selectable image into CBF files
02000	Put selected results into root file

**Table 4:** Analysis Processors

Number	Purpose
00200	Scalar value of 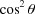 from 2D result
00201	Angular distribution from a 2D result (interpolating)
00203	Local image background using median box
00208	Find Bragg peaks in a 2D result using signal-to-noise ratio without outliers
00205	Display peaks found in a 2D result
00300	Single-particle detection
00311	Autocorrelation of image in Cartesian coordinates
00330	Generate calibration data from raw 2D results
00331	Generate gain calibration from 2D results
00332	Generate hot pixel map from 2D results
00333	Generate common mode background level
00243	Apply a mask to a 2D result, set the masked values to a user-defined value

## Appendix A List of selected Processors

Tables 1 ▸–4 ▸ ▸ ▸ show a selection of the available Processors already existing in *CASS*. The modules in Table 1 ▸ will function on the results of other Processors, while the modules in Table 2 ▸ are used to retrieve data stored in a CASSEvent. The results of interest can be written for display or further analysis using Processors in Table 3 ▸. More specialized analysis on other results or data stored in the CASSEvents directly can be performed using modules listed in Table 4 ▸.
